# Late Cretaceous Vicariance in Gondwanan Amphibians

**DOI:** 10.1371/journal.pone.0000074

**Published:** 2006-12-20

**Authors:** Ines Van Bocxlaer, Kim Roelants, S.D. Biju, J. Nagaraju, Franky Bossuyt

**Affiliations:** 1 Biology Department, Unit of Ecology and Systematics, Vrije Universiteit Brussel Brussels, Belgium; 2 Centre for Environmental Management of Degraded Ecosystems (CEMDE), School of Environmental Studies, University of Delhi Delhi, India; 3 Laboratory of Molecular Genetics, Centre for DNA Fingerprinting and Diagnostics Hyderabad, India; Max Planck Institute for Evolutionary Anthropology, Germany

## Abstract

Overseas dispersals are often invoked when Southern Hemisphere terrestrial and freshwater organism phylogenies do not fit the sequence or timing of Gondwana fragmentation. We used dispersal-vicariance analyses and molecular timetrees to show that two species-rich frog groups, Microhylidae and Natatanura, display congruent patterns of spatial and temporal diversification among Gondwanan plates in the Late Cretaceous, long after the presumed major tectonic break-up events. Because amphibians are notoriously salt-intolerant, these analogies are best explained by simultaneous vicariance, rather than by oceanic dispersal. Hence our results imply Late Cretaceous connections between most adjacent Gondwanan landmasses, an essential concept for biogeographic and palaeomap reconstructions.

## Introduction

Ever since Alfred Wegener's theory in 1915, biologists have had vicariance at their disposal as a mechanism to logically explain transoceanic distributions in plants and animals. Meta-analyses of area cladograms [1] show that many animal phylogenies match the sequential break-up of Gondwana and thus support this hypothesis. However, there is an increasing number of Southern Hemisphere taxa for which divergence time estimates do not readily fit the temporal framework of fragmentation [2], [3]. This lack of concordance has sometimes led to the conclusion that the distribution of these organisms resulted from overseas dispersal events [e.g. 4], rather than from a vicariant history. Although transoceanic dispersal may have played an important role in several taxa [5], the possibility that terrestrial connections have existed longer than assumed under traditional geological models, has received limited attention in the dispersal-vicariance debate. Yet, if theories of prolonged connections [6], intervening landmasses [2], [3], or a completely enclosed Pacific Basin [7] in the Late Cretaceous would prove to be correct, several of the younger phylogenies might still be explained by Gondwanan vicariance.

While geology is typically used to calibrate the Tree of Life, molecular timetrees of organisms with no (or very limited) oceanic dispersal capabilities in turn have the property that they can test for the presence of associations among tectonic plates. Indeed, under this premise, divergence times between continent-scale endemic groups provide a maximum age for the actual separation of landmasses (i.e., the disappearance of a terrestrial connection). Amphibians are an extremely good model for such studies, because they are notoriously salt-intolerant and, as a rule, oceans provide an effective barrier against their transoceanic dispersal [5]. An exceptional case of short-distance transoceanic dispersal to the Comores has been shown [8], but amphibians do not occur on other oceanic islands and there is no evidence for long-distance overseas dispersal in these animals. Thus it is surprising that molecular clock analyses date the origin of two species-rich Gondwanan groups, Natatanura and Microhylidae, well in the Cretaceous [8]–[11]. If correct, the subsequent diversification of the major lineages within these clades cannot be congruent with initial Gondwanan break-up, which is traditionally depicted in the Early Cretaceous (145–100 mya). To address this controversy, and to test the hypothesis of prolonged biotic interchange between Gondwanan landmasses, we checked for phylogenetic and temporal concordance between and within Microhylidae and Natatanura, using 3.1 and 5.6 kb of sequence data for the major lineages, respectively [12].

## Results

**Figure 1:**
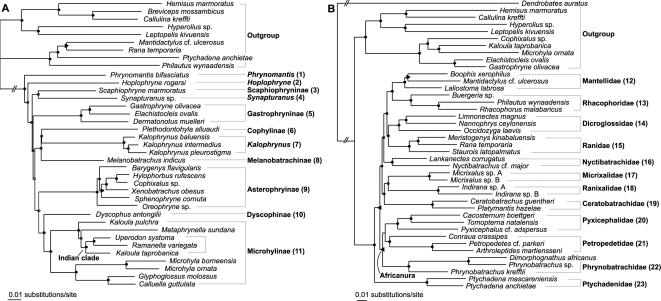
Maximum likelihood phylograms for (**A**) Microhylidae (-lnL = 24126.41696) and (**B**) Natatanura (-lnL = 53106.71363). Branch support is indicated as follows: black circles: ML bootstrap support (BS)≥75% and Bayesian posterior probability (PP)≥0.95; triangle pointing to the right: BS<75%, PP≥0.95; triangle pointing to the left: BS≥75%, PP<0.95; white circles: BS<75% and PP<0.95. Higher taxon names follow Frost et al. [13], with the exception of removal of *Hoplophryne* from Melanobatrachinae, and recognition of Ranixalidae.

Our Maximum likelihood (ML) and Bayesian phylogenetic trees differ from Frost et al.'s ‘Amphibian Tree of Life’ [13] in some important biogeographic aspects (Figure 1, Table 1). First, in Microhylidae, we recover Dyscophinae (not including the Asian *Calluella*) as the closest relatives of Microhylinae (*i.e.*, a Madagascar-Eurasia/India sister-relationship) (Figure 1) instead of its alternative position as sister group of Asterophryinae. The alternative of Scaphiophryninae being the sistergroup of Microhylinae [13], although implying the same biogeographic pattern, is rejected by our data (Table 1, hypotheses 1–3). Second, our analyses do not support a sister-clade relationship between the African genus *Hoplophryne* and *Ramanella* from the Indian subcontinent (Table 1, hypothesis 4), but recover the latter in an unexpected clade endemic to the Indian Subcontinent (Figure 1A, Indian clade). Third, our analyses show that Melanobatrachinae and Microhylinae are restricted to India and India/Eurasia, respectively, and that their assumed relatives in Africa and South America represent separate lineages (Table 1, hypothesis 5 and 6). Fourth, *Kalophrynus* as sistergroup of all remaining microhylids, which would imply a basal position for a strictly Eurasian lineage, is also rejected by our data (Table 1, hypothesis 7). Finally, we find strong support for an Indian endemic clade composed of the genera *Indirana* and *Micrixalus* (Figure 1B). The nested position of *Indirana* within six hierarchic African clades, as found by Frost et al. [13] is strongly rejected (Table 1, hypotheses 8–13), identifying Africanura as an exclusively African radiation (Figure 1B). All of our alternatively recovered relationships favor simpler biogeographic scenarios than previous hypotheses and complete the evidence that amphibians experienced continent-scale endemic radiations [11] as the result of a general vicariance history with little overseas dispersal.

**Table 1:**
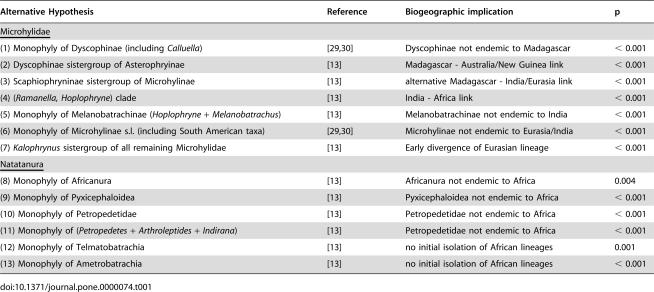
Bayesian test for alternative hypotheses (and their associated biogeographic implication) of relationships in Microhylidae and Natatanura.

Alternative Hypothesis	Reference	Biogeographic implication	p
Microhylidae			
(1) Monophyly of Dyscophinae (including *Calluella*)	[29], [30]	Dyscophinae not endemic to Madagascar	< 0.001
(2) Dyscophinae sistergroup of Asterophryinae	[13]	Madagascar - Australia/New Guinea link	< 0.001
(3) Scaphiophryninae sistergroup of Microhylinae	[13]	alternative Madagascar - India/Eurasia link	< 0.001
(4) (*Ramanella, Hoplophryne*) clade	[13]	India - Africa link	< 0.001
(5) Monophyly of Melanobatrachinae (*Hoplophryne* + *Melanobatrachus*)	[13]	Melanobatrachinae not endemic to India	< 0.001
(6) Monophyly of Microhylinae s.l. (including South American taxa)	[29], [30]	Microhylinae not endemic to Eurasia/India	< 0.001
(7) *Kalophrynus* sistergroup of all remaining Microhylidae	[13]	Early divergence of Eurasian lineage	< 0.001
Natatanura			
(8) Monophyly of Africanura	[13]	Africanura not endemic to Africa	0.004
(9) Monophyly of Pyxicephaloidea	[13]	Pyxicephaloidea not endemic to Africa	< 0.001
(10) Monophyly of Petropedetidae	[13]	Petropedetidae not endemic to Africa	< 0.001
(11) Monophyly of (*Petropedetes + Arthroleptides* + *Indirana*)	[13]	Petropedetidae not endemic to Africa	< 0.001
(12) Monophyly of Telmatobatrachia	[13]	no initial isolation of African lineages	0.001
(13) Monophyly of Ametrobatrachia	[13]	no initial isolation of African lineages	< 0.001

All hypotheses either represent long-established taxa [29]–[30] or have been proposed recently [13].

**Figure 2:**
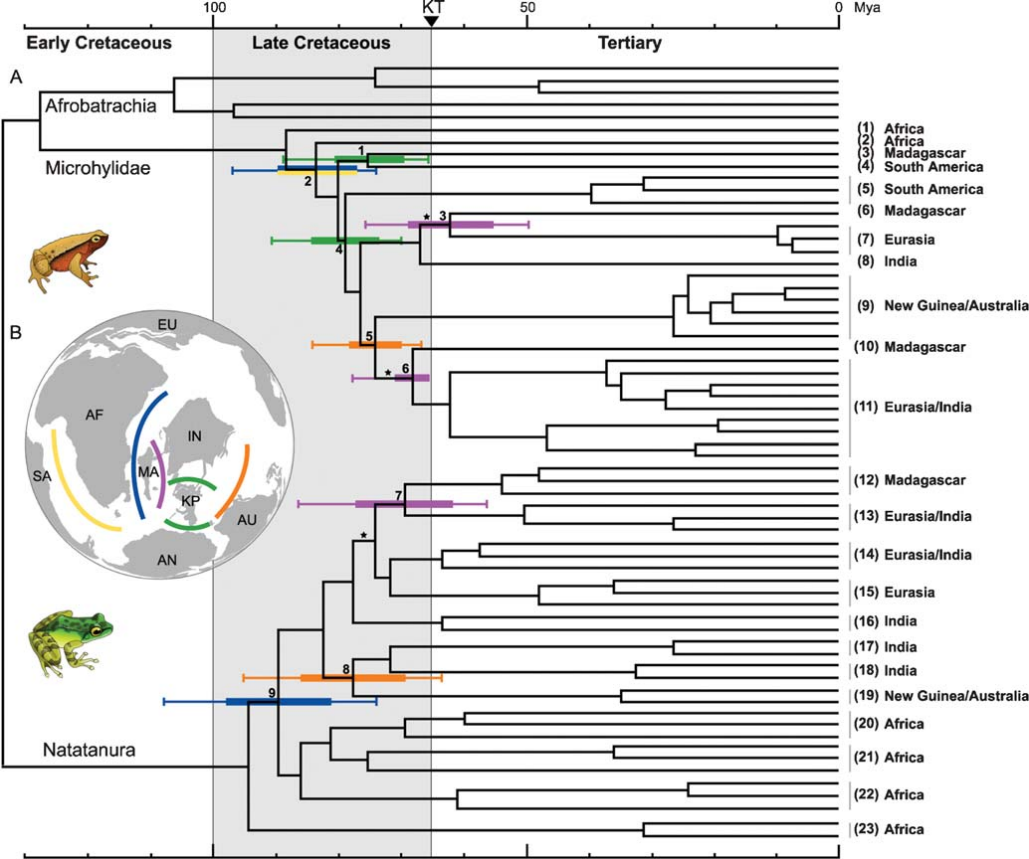
Late Cretaceous vicariance in Microhylidae and Natatanura. (**A**) Molecular timetree (TK method, all calibration points except G [12]). Horizontal colored bars and lines at internal nodes (Standard deviation and 95% credibility interval, respectively) indicate vicariance events reconstructed by DIVA-analyses, and interpreted as follows: orange: Australia <–> Indo-Madagascar; yellow: Africa <–> South America; blue: Africa <–> Indo-Madagascar; purple: Madagascar <–> India (Seychelles); green: S. America-Antarctica <–> Indo-Madagascar (the intervening Kerguelen Plateau being involved). The latter splits in our timetree are interpreted as vicariance events between the Kerguelen plateau and Antarctica or Indo-Madagascar [12]. The branches denoting the latest colonization of Eurasia, as reconstructed by DIVA, are indicated by an asterisk. Numbers at terminals correspond to taxon numbers in figure 1. (**B**) Late Cretaceous Gondwana, with indication of corresponding geological break-ups. Abbreviations: AF = Africa, MA = Madagascar, IN = India, EU = Eurasia, SA = South America, AN = Antarctica, AU = Australia-New Guinea, KP = Kerguelen Plateau.

To estimate the age of early divergences in Microhylidae and Natatanura, we combined both clades in a single data matrix and performed relaxed molecular clock analyses using two independent methods and multiple combinations of calibration points [12]. Our age estimates are largely congruent with recent studies focusing on deeper anuran divergences or on natatanuran relationships [8]–[11]. Importantly, all our timetrees placed the 95% confidence intervals of early divergences in both groups in the Late Cretaceous (Figure 2A, gray block) and Early Tertiary (Table 2). These results indicate that each of the endemic groups became isolated long after the traditionally depicted break-up events [14].

**Table 2:**
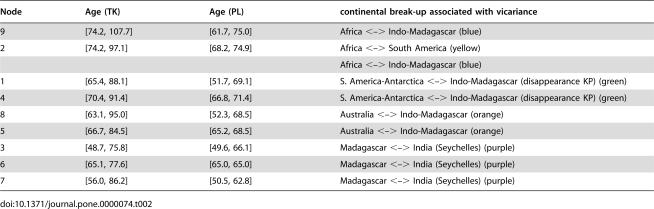
Dating estimates (mya) for vicariance events in the early evolution of Microhylidae and Natatanura.

Node	Age (TK)	Age (PL)	continental break-up associated with vicariance
9	[74.2, 107.7]	[61.7, 75.0]	Africa <–> Indo-Madagascar (blue)
2	[74.2, 97.1]	[68.2, 74.9]	Africa <–> South America (yellow)
			Africa <–> Indo-Madagascar (blue)
1	[65.4, 88.1]	[51.7, 69.1]	S. America-Antarctica <–> Indo-Madagascar (disappearance KP) (green)
4	[70.4, 91.4]	[66.8, 71.4]	S. America-Antarctica <–> Indo-Madagascar (disappearance KP) (green)
8	[63.1, 95.0]	[52.3, 68.5]	Australia <–> Indo-Madagascar (orange)
5	[66.7, 84.5]	[65.2, 68.5]	Australia <–> Indo-Madagascar (orange)
3	[48.7, 75.8]	[49.6, 66.1]	Madagascar <–> India (Seychelles) (purple)
6	[65.1, 77.6]	[65.0, 65.0]	Madagascar <–> India (Seychelles) (purple)
7	[56.0, 86.2]	[50.5, 62.8]	Madagascar <–> India (Seychelles) (purple)

For both the TK- and PL-method, the ages represent the 95% confidence intervals of the analysis that includes all calibration points except G [12]. Node numbers and colors associated with break-up correspond with figure 2. KP, Kerguelen Plateau.

We used Dispersal-Vicariance analyses [12] to reconstruct the biogeography of Late Cretaceous and Early Tertiary nodes in Natatanura and Microhylidae. This method has the advantage of not being constrained by a particular break-up sequence, and thus does not favor the traditional model of Gondwanan fragmentation *a priori*. Our analyses suggest six vicariance events among the following adjacent Gondwanan landmasses (Figure 2, Table 2, nodes 1, 2, 4, 5, 8, 9): Indo-Madagascar *versus* Africa (blue), Indo-Madagascar *versus* Australia-New Guinea (orange), Africa *versus* South America (yellow), and South America-Antarctica *versus* Indo-Madagascar (over the Kerguelen plateau) (green). Most of our inferred 95% credibility intervals for the divergence time estimates of these nodes show no overlap with the temporal windows of traditional plate-tectonic models, which indicate that Indo-Madagascar separated from Africa and Antarctica-Australia-New Guinea in the Early Cretaceous (165–121 mya and 130–120 mya, respectively), and Madagascar from India about 88 mya [14]. However, the strong temporal overlap between 95% age intervals of equally reconstructed splits (compare the range of identically colored intervals in Figure 2) suggests that they were shaped by the same paleogeographic events and hence, that they reflect the actual separation of the corresponding landmasses.

## Discussion

Under a strict interpretation of the DIVA results, the Madagascar-Eurasia/India relationships require three contemporary dispersals (Figure 2, purple) directly from Madagascar to Eurasia (i.e., transoceanic, not using India). However, accretion of landmasses, like vicariance, also allows multiple groups to simultaneously depict the same change in their distribution [15], [16]. We suggest that this contemporary, repeated pattern of area relationships is a case of ‘mass coherent dispersal’ [15], [16], resulting from taxa on isolated India (or Indo-Madagascar) dispersing to Eurasia after contact [17]. Under this interpretation, the Madagascar-Eurasia splits in fact reflect the vicariance between Madagascar and India (Figure 2, purple). The three extinctions needed on India to accept this scenario can be easily explained by mass extinction [12], given that the Deccan Traps flooded large parts of the subcontinent around the Cretaceous-Tertiary (KT) boundary.

Our combined phylogenetic, temporal, and biogeographic evidence in two frog clades is indicative of Late Cretaceous terrestrial connections between most adjacent Gondwanan landmasses. Although this contrasts with traditional plate-tectonic models [14], various alternative hypotheses have been proposed that could accommodate our results. First, our phylogenies suggest that African frog lineages in both groups became isolated first (*i.e.*, an ‘Africa first’ scenario [2], [18]). Vicariance events in the early Late Cretaceous (Table 2, nodes 2 and 9) are consistent with the prolonged existence of a South America-Africa connection [18], as well as the postulation of a ‘Central Corridor’ between Africa and Indo-Madagascar until ∼90 mya [6]. Second, the Kerguelen Plateau (Figure 2B) and Gunnerus Ridge, that may have connected Indo-Madagascar and Antarctica until ∼80 mya [19]–[22] provide an explanation for microhylid vicariances between South America and Indo-Madagascar. Third, paleontological and paleogeographic studies have recently suggested that both the Tethys Sea and Pacific Basin may have been much smaller and more enclosed by surrounding continents than previously assumed [7], [23]. As a consequence, India may have accreted to Eurasia already in the Late Cretaceous, while it was still connected to Madagascar over the Seychelles Plateau [22], [24]. This would account for the triple, deeply nested Madagascar-Eurasia/India relationships recovered in our phylogenies.

A relaxed scenario for Gondwanan break-up helps to explain several observations in the fossil record. For example, several mammalian, dinosaur, and crocodilian taxa seem to have attained widespread distributions in Gondwana only by the Late Cretaceous [2], [18], [20]. Furthermore, several palaeontological studies have suggested close affinities of South East Asian taxa with Indian, Madagascan, and Australia-New Guinean taxa in the Late Cretaceous [25]–[27]. Although fossils inevitably underestimate the true age of divergences, these observations provide a better fit to a model that allows both prolonged biotic interchange between landmasses of Gondwanan origin, and a Late Cretaceous connection of Indo-Madagascar and Australia-New Guinea with South East Asia. Landmasses that experienced recognized mass extinctions, such as Antarctica, the Kerguelen Plateau, and India, must have played a central role in Late Cretaceous range extensions. Future palaeobiogeographical research on biota of Southern Hemispere origin will clearly benefit from key fossil evidence from these regions.

## Materials and Methods

### Sampling

Phylogenetic relationships within Microhylidae and Natatanura were estimated using independently assembled data sets, each encompassing the major lineages within one family. The microhylid data set contains 2865 unambiguously aligned nucleotides, sampled from three nuclear protein-coding genes (*Cxcr-4, Ncx-1, Rag-1*) and one mitochondrial gene (*16S rDNA*) (Table S1). The ingroup is composed of 28 microhylid species, covering all previously and currently recognized subfamilies within this family [13], [28]–[31] (Table S2). Four natatanuran and five afrobatrachian species served as outgroup. The natatanuran data set contains 4446 unambiguously aligned nucleotides, combining the same three nuclear gene fragments mentioned above with coding regions of two additional nuclear genes (*Tyr* and *Rhod*), and an extended mitochondrial fragment (*12S rDNA*+*tRNA(Val)*+*16S rDNA*) (Table S3). The ingroup comprises 31 natatanuran species, while one dendrobatid, four afrobatrachian and five microhylids were selected as outgroup (Table S4). Genbank accession numbers are summarized in table S5 (Microhylidae), table S6 (Natatanura), and table S7 (outgroup species).

### Phylogenetic analyses

Phylogenetic relationships were reconstructed for both data sets using Bayesian analyses and maximum likelihood (ML) searches (Figure 1). All analyses implemented the GTR+G+I model of DNA substitution, which received the best Akaike information score by Modeltest 3.06 [32]. Bayesian analyses were performed with MrBayes 3.1.2 [33], using a mixed model according to a gene-based data partition. Two runs of four MCMC chains each were executed in parallel for five million generations, with a sampling interval of 1000 generations and a burn-in corresponding to the first one million generations. Convergence of the parallel runs was confirmed by split frequency standard deviations (<0.001), and by potential scale reduction factors (PSRF; ∼1.0) for all model parameters. Posterior probabilities for clades were obtained by combining the post-burn-in trees from parallel runs in a single consensus tree. Heuristic ML searches were performed with PAUP* 4.0b10 [34] and involved multiple rounds of TBR branch swapping, alternatively starting from Bayesian consensus trees or from ML trees estimated by the program Phyml 2.4.1 [35]. All searches implemented empirical nucleotide frequencies and fixed rate parameters, estimated in advance from the starting trees. Clade support under the likelihood criterion was assessed by analyzing 1000 nonparametric bootstrap replicates using Phyml. Bayesian posterior probabilities for alternative microhylid and natatanuran phylogenetic hypotheses [13], [29]–[30] were estimated by screening the post-burn-in trees sampled by MrBayes using topological constraint filters in PAUP*.

### Tree calibration and dating

We performed dating analyses using two different relaxed molecular clock methods, which have recently been demonstrated to be the least sensitive to taxon sampling [36], and which have complementary advantages and limitations. Thorne & Kishino's (TK) method [37] accommodates unlinked rate variation across different loci (a ‘multi-gene’ approach), allows the use of time constraints on multiple divergences, and uses a Bayesian MCMC approach to approximate the posterior distribution of divergence times and rates, but uses an F84+G model (or a nested variant) for branch length estimation, and fails to incorporate phylogenetic uncertainty in the posterior distribution. Sanderson's penalized likelihood (PL) method [38] allows branch length estimation using more complex DNA substitution models (*e.g.*, GTR+G+I) and the use of a posterior tree set to estimate credibility intervals (CI), but necessarily averages rate variation over all loci (a ‘supergene’ approach), and requires a time-consuming cross-validation method to determine optimal rate smoothing penalty parameters. To maximize the overall accuracy of our dating estimates, we sought to obtain an optimal phylogenetic coverage of calibration points across our tree (Figure S1, Table S8). However, to minimize the risk of over-constraining the resulting timetree, we used them only as minimum time constraints. The results of TK- and PL-analyses with different combinations of calibration points are listed in Table S9. Removal of individual time constraints in most cases resulted in highly congruent dating estimates with respect to the total set of calibration points (Figure S2), with the exception of two paleogeographic calibration points. However, these estimates resulted in a *younger* age for reconstructed natatanuran and microhylid vicariance events, and thus would imply an even larger discrepancy with currently accepted models for Gondwana break-up.

### Biogeographic analyses

To quantify vicariance events in the early diversification of both groups, we estimated ancestral distributions for ingroup nodes using dispersal-vicariance analyses performed by the program DIVA 1.1 [39]. All ingroup taxa were coded for their present distribution across six biogeographic units (five of which represent separate fragments of Gondwana): Africa, Madagascar, India, South America, Australia-New Guinea and Eurasia (Table S10, Figures S3, S4). To simplify the analyses, we saved an extra biogeographic unit by coding the North American *Gastrophryne olivacea* as a South American taxon, because of its nested position within the neotropical clade Gastrophryninae.

## Supporting Information

Materials and Methods S1 (0.17 MB DOC)

Figure S1 Phylogenetic tree used for the divergence time analyses. The natatanuran and microhylid clades are resolved according to the ML topologies obtained from their respective data sets (Figure 1). Outgroup divergences are resolved according to previous phylogenetic evidence (see Methods). Numbers at ingroup nodes are cross-referenced in Table S9, Letters indicate calibration points.
(11.84 MB TIF)

Figure S2 Effect of excluding individual calibration points on divergence time estimates. Letters refer to excluded calibration points and correspond to Tables S8 and S9. The bars represent mean age differences, obtained with the TK-method (orange) and the PL method (blue), compared to the analysis including all calibration points except G.
(5.34 MB TIF)

Figure S3 DIVA-reconstruction of ancestral distribution areas. Letter codes at internal nodes correspond to landmasses depicted on the inset globe.
(8.35 MB TIF)

Figure S4 DIVA-reconstruction of ancestral distribution areas, assuming three divergences on Indo-Madagascar. Reconstruction of ancestral distributions under the assumption that nodes 3, 6 and 7 represent vicariance events related to India-Madagascar break-up. Letter codes at internal nodes correspond to landmasses depicted on the inset globe.
(8.35 MB TIF)

Table S1 Summary of the sequence data for all sampled nuclear and mitochondrial gene fragments and the total dataset in Microhylidae.
(0.31 MB DOC)

Table S2 Taxa included in the Microhylidae dataset, with voucher numbers and origin.
(0.34 MB DOC)

Table S3 Summary of the sequence data for all sampled nuclear and mitochondrial gene fragments and the total dataset in Natatanura.
(0.31 MB DOC)

Table S4 Taxa included in the Natatanura dataset, with voucher numbers and origin.
(0.34 MB DOC)

Table S5 Taxa with GenBank accession numbers of homologous gene fragments for microhylid species.
(0.34 MB DOC)

Table S6 Taxa with GenBank accession numbers of homologous gene fragments for natatanuran species.
(0.36 MB DOC)

Table S7 Taxa with GenBank accession numbers of homologous gene fragments for outgroup species.
(0.37 MB DOC)

Table S8 Calibration points used in this study.
(0.34 MB DOC)

Table S9 Divergence time estimates (standard deviation; 95% credibility intervals) for all ingroup nodes in our dating-tree, obtained with different calibration points and dating methods.
(0.16 MB XLS)

Table S10 Dating estimates (mya) for Microhylidae and Natatanura, with DIVA reconstruction of vicariance events in their early evolution. For both the TK- and PL-method, the ages represent the 95% confidence intervals of the analysis that includes all calibration points except G. Vicariance (1) represents the strict analysis; Vicariance (2) represents the analysis with the three India-Eurasia splits constrained as India-Madagascar. The two DIVA reconstructions represent the actual distribution of the frogs, while the associated continental break-up is the presumed geological unit at the moment of break-up, based on our divergence times. Node numbers and colors associated with break-up correspond with figure 2. Afr = Africa, Ind = India, SAm = South America, Mad = Madagascar, Aus = Australia-New Guinea, Eur = Eurasia, KP = Kerguelen Plateau.
(0.33 MB DOC)
