# Supplementary material for: Late Cretaceous Vicariance in Gondwanan Amphibians
Source: PLoS One. 2006 Dec 20;1(1):e74. doi: 10.1371/journal.pone.0000074 (PMC1762348; doi:10.1371/journal.pone.0000074)
Supplement: Materials and Methods S1 — (0.17 MB DOC) [file pone.0000074.s001.doc]

# Supporting Information

Late Cretaceous Vicariance in Gondwanan Amphibians

Ines Van Bocxlaer, Kim Roelants, S.D. Biju, J. Nagaraju and Franky Bossuyt*

*To whom correspondence should be addressed. E-mail: fbossuyt@vub.ac.be

**This PDF file includes:**

### Material and Methods

# 1. Phylogenetic reconstructions

# 2. Divergence time estimation

2.1 Dating methods

2.2 Calibration points

2.3 Comparison and Evaluation of the divergence time analyses

3. Biogeographic reconstructions

4. References

**1. Phylogenetic reconstructions**

Amphibian taxonomy is currently in a state of flux, but this study follows the classification of Frost et al.’s recently published ‘Amphibian Tree of Life’ [1], which is based on the largest body of phylogenetic evidence hitherto published. Because this taxonomy differs markedly from previously proposed classifications, we also make reference to Dubois’ ‘Amphibia mundi’ [2], which retained more consistency with long-established systematic arrangements [3, 4].

DNA sequences for both data sets were either retrieved from previous studies [5-8] or newly obtained via whole-genome DNA extraction, PCR amplification, gel extraction and cycle-sequencing along both strands. Sequences of used primers are provided elsewhere [7, 9, 10]. Alignments for all individual gene fragments were created with ClustalX 1.81 [11] and manually corrected in MacClade 4.06 [12]. Phylogenetic analyses [13-19] are explained in detail in the Materials and Methods section of the main text.

**2. Divergence time estimation**

*2.1. Dating methods*

All dating analyses were performed on a single combined data set. To increase the number of available calibration points, homologous sequences from the microhylid and natatanuran data sets (*i.e.*, Cxcr-4, Ncx-1, Rag-1 and 16s rDNA) were realigned with the addition of 15 non-ranoid frogs, five salamanders, four amniotes and a chimeric taxon composed of two fishes (2639 unambiguously aligned nucleotides). We assembled a corresponding input tree by combining the separately estimated ML topologies for Microhylidae and Natatanura with phylogenetic information from previous studies [1,10,20,21] (Figure S1).

We performed dating analyses using two different relaxed molecular clock methods, which have recently been demonstrated to be the least sensitive to taxon sampling [22], and which have complementary advantages and limitations. Thorne & Kishino’s (TK) method [23] accommodates unlinked rate variation across different loci (a ‘multi-gene’ approach), allows the use of time constraints on multiple divergences, and uses a Bayesian MCMC approach to approximate the posterior distribution of divergence times and rates, but uses an F84+G model (or a nested variant) for branch length estimation, and fails to incorporate phylogenetic uncertainty in the posterior distribution. Sanderson’s penalized likelihood (PL) method [24] allows branch length estimation using more complex DNA substitution models (e.g., GTR+G+I) and the use of a posterior tree set to estimate credibility intervals (CI), but necessarily averages rate variation over all loci (a ‘supergene’ approach), and requires a time-consuming cross-validation method to determine optimal rate smoothing penalty parameters. To investigate the sensitivity of divergence time estimates to these differences, we analyzed our ML tree with both methods.

*TK analyses*.—Analyses with the TK method were performed with the MULTIDIVTIME software (online available at http://statgen.ncsu.edu/thorne/multidivtime.html). DNA substitution branch lengths were estimated per gene fragment with the program ESTBRANCHES, using an F84+G model with parameters estimated by PAUP*. Proper approximation of the optimal branch lengths was verified by comparing the resulting log-likelihood values with those estimated by PAUP*. Optimized branch lengths with their variance-covariance matrices were used as input for the program MULTIDIVTIME, which calculates 95% credibility intervals for node ages, based on relaxed-clock model priors and calibration points (see further). We set the following priors for the relaxed-clock model:

*Ingroup root age.*—The priors for the mean and standard deviation of the ingroup root age, *rttm* and *rttmsd* were set to equivalents of 344 million years ago (Mya), and 20 million years (Myr), respectively. This defines a fairly broad prior distribution for the split between amniotes and living amphibians, that covers both the Viséan (Early Carboniferous) age estimates for the crown-tetrapod origin based on recent stratigraphic analyses of the fossil record [25,26], and the Famennian (Late Devonian) age estimates implied by earlier postulated phylogenies [27] and molecular clock analyses [20,28,29].

*Ingroup root rate.*—The priors for the mean and standard deviation of the ingroup root rate, *rtrate* and *rtratesd*, were both set to 0.122 (substitutions per site per 100 Myr). These values were based on the median of the substitution path lengths between the ingroup root and each terminal, divided by *rttm* (as suggested by the author).

*Brownian motion model*.—The priors for the mean and standard deviation of the Brownian motion constant, *brownmean* and *brownsd*, were both set to 0.4, specifying a relatively flexible prior.

The single MCMC chain was run for 1.1 million generations, with a sampling frequency of one per 100 generations and a burn-in corresponding to the first 100,000 generations. Generation-series plots of sampled divergence times and repeated analyses confirmed that this sampling configuration was sufficient to reach posterior stationarity of divergence time estimates.

*PL analyses*.-—The rooted ML phylogram, with branch lengths estimated by PAUP* under a GTR+G+I model, was used as input tree for the program R8S 1.70 [30]. Analyses were performed with a truncated-Newton (TN) optimization algorithm as suggested by the author. The optimal rate-smoothing penalty parameter was determined by the statistical cross-validation method implemented in R8S. A first cross-validation series compared rate-smoothing parameters across a log10-scale from –3 to 8 with an increase of 0.1. Analyses using the resulting optimal rate-smoothing penalty values were started from 5 different random combinations of divergence times (the *num_time_guesses* option) and with a gradient check of the objective function at solution (the *checkgradient* option). Credibility intervals on these divergence time estimates were obtained by: (*i*), generating 200 nonparametric bootstrap replicates of the original data set with the program SeqBoot of the PHYLIP 3.6b software package [31]; (*ii*), estimating branch lengths for our ML-topology on each of these replicate data sets using PAUP*; (*iii*), repeating the PL-analyses for each of the resulting 200 replicate phylograms, using the same calibration configuration but individually estimated optimal rate smoothing parameters.

*2.2. Calibration points*

It has been argued that the use of multiple calibration points would provide overall more realistic divergence time estimates, as single or few calibration points are likely to result in high estimation errors for distantly related nodes [22,32,33]. To maximize the overall accuracy of our dating estimates, we sought to obtain an optimal phylogenetic coverage of calibration points across our tree. However, to minimize the risk of over-constraining the resulting timetree, we used them only as minimum time constraints. Because the amphibian fossil record is notoriously poor, it is likely that the fossil age of some lineages largely underestimates their true age [34]. In such case, rather than forcing nearby nodes to be underestimated as well, a minimum time constraint will not contribute much to the inferred age estimates, and the result will be mainly determined by the molecular data (conditional on other calibration points).

Examination of the fossil and tectonic records in light of our taxon sampling yielded minimum time constraints for eight amphibian divergences. These were used in combination with a conservative age interval (imposing a minimum *and* a maximum) for the amniote crown-group (see below). The PL analyses additionally require constraints on the ingroup root (the split between amniotes and living amphibians). We used the interval 326–383.5 Mya for this divergence, which brackets the Late Devonian–Early Carboniferous (Viséan) period, and shows strong overlaps with the prior distribution used in the TK analyses. An overview of all calibration points is provided in Table S8. Some of them require more explanation and are discussed below:

*Calibration point D.*—The minimum of 145.5 Mya for the split between cryptobranchid and hynobiid salamanders is based on the fossil *Chunerpeton tianyiensis*, recovered from the Inner Mongolian Daohugou Beds [35]. Although the authors report a Bathonian [Middle-Jurassic age for these Beds as part of the Jiulongshan Formation [36]], they are often considered to be of Late-Jurassic/Early-Cretaceous age [as part of the Yixian Formation or Jehol Group [37,38]]. Therefore, to provide a more conservative minimum age constraint, we set for the Jurassic/Cretaceous boundary, at 145.5 Mya.

*Calibration points F and G.*—Because recent studies have emphasized the importance of internal calibration points in dating analyses (i.e. nested within the clade of interest) [33,39,40], we imposed minimum time constraints within both Natatanura and Microhylidae. Natatanura have a relatively rich Eurasian fossil record from the Early Tertiary on (see Calibration point H), but reliable microhylid fossils are currently unknown [apart from a single Miocene finding from Florida [41], which is probably too young to affect our estimates]. As an alternative, we performed TK- and PL analyses using a minimum time constraint of 65 Mya on either: (*i*), the divergence between Madagascan *Dyscophus* and Asian Microhylinae (Calibration point F), or (*ii*), the split between Madagascan Cophylinae and Asian *Kalophrynus* (Calibration point G). Given the apparent Gondwanan origin of microhylid frogs, the limited capacity of amphibians to cross oceanic barriers, and the fact that Madagascar and Eurasia were never directly connected, the dispersal of Microhylidae to Eurasia is best explained by the Indian subcontinent, during or after its break-up from Madagascar and collision with Asia [i.e. an ‘Out-of-India’ scenario, similar to natatanuran frogs [42] and ichthyophiid caecilians [43]. The *Dyscophus*–Microhylinae and *Kalophrynus*–Cophylinae splits then, would at least have happened at, or prior to the subcontinent’s break-up from Madagascar. Although both landmasses are generally assumed to have separated ~88 Mya [44], we use 65 Mya as a more conservative minimum to accommodate the existence of any Indo-Madagascan land connection until the end of the Cretaceous [45-47]. The effect of using neither of these two calibration points was evaluated by cross validation analyses.

*Calibration point H*.—A minimum of 28.5 Mya for the stem origin of *Rana* is based on the earliest fossil remains of green water frogs (*Pelophylax*) in Europe, dated at MP 22 (Rupelian, Early Oligocene) [48]. Although molecular phylogenetic studies have shown that *Pelophylax* and the brown frogs of the subgenus *Rana* represent separate lineages, they diverged within Ranidae [Raninae *sensu* [2]] after the divergence of their common ancestor from *Meristogenys* [5], en thus represent the same lineage in our timetree.

*Calibration point I*.—The interval 306.1–332.3 Mya for the split between Diapsida (including birds and lizards) and Synapsida (including mammals) represents a fair relaxation of the often-used ‘310-Mya-calibration’ for this divergence event [29,49]. Because the accuracy of this point estimate has recently attracted criticism from both paleontologists and molecular biologists [50-52], the currently applied interval has been proposed as a conservative correction, based on the age and diversity of both crown- and stem-amniotes [53].

A first round of analyses was performed using calibration points A–F + H–I (excluding calibration point G) and A–E + G–I (excluding calibration point F). To evaluate the individual influence of all individual calibration points, we performed seven additional TK- and PL-analyses (without calibration point G), each time excluding a different calibration point (*i.e.*, excluding one of calibration points A–E, H–I). This method is similar to the fossil cross validation procedure proposed by Near and Sanderson [54]). Because calibration points F and G are based on the same paleogeographic event, we also performed an analysis excluding both. To obtain a measure of the effect of removing time constraints, we inferred for each analysis the average difference between the newly obtained divergence age and the one obtained by including all calibration points.

*2.3 Comparison and Evaluation of the divergence time analyses*

The results of TK- and PL-analyses with different combinations of calibration points are listed in Table S9. All of them place the early diversification of Microhylidae and Natatanurana in the Late Cretaceous-Early Tertiary. Separate analyses using either calibration point F (*Dyscophus*-Microhylinae split set at > 65 Mya) or G (*Kalophrynus*-Cophylinae split set at > 65 Mya) resulted in very similar age estimates, both within Microhylidae and Natatanura (Table S9, compare Estimates 1 and 2). In addition, use of calibration point F situated the *Kalophrynus*-Cophylinae split at 61.8 ± 6.8 Mya, closely approximating calibration point G. Likewise, use of calibration point G situated the *Dyscophus*-Microhylinae split at 66.1 ± 5.8 Mya, indicating a strong compatibility of both calibration points. PL-analyses produced overall slightly younger time estimates for microhylid and natatanuran divergences than those inferred by the TK-method (absolute: 5.3 ± 4.9 Myr younger; relative: 6.9 ± 9.5 % younger). In addition, our divergence time estimates (Table S9) show relatively high congruence with those observed in previous studies, based on alternative data set compositions and calibration strategies. [8,20,28,55,56].

Removal of individual time constraints in most cases resulted in highly congruent dating estimates with respect to the total set of calibration points (Figure S2), with the exclusion of calibration points F + G (on the *Dyscophus*–Microhylinae and *Kalophrynu*s–Cophylinae splits) yielding the overall largest reduction in divergence age (TK-method: 9.8 ± 5.1 Myr younger; PL-method: 8.2 ± 5.9 % younger). When none of the two paleogeographic calibration points were included, several of the reconstructed natatanuran and microhylid vicariance events were shifted towards the Early Tertiary, resulting in an even larger discrepancy with currently accepted models for Gondwana break-up.

# **3. Biogeographic reconstructions**

To quantify vicariance events in the early diversification of both groups, we estimated ancestral distributions for ingroup nodes using dispersal-vicariance analyses performed by the program DIVA 1.1 [57].

An exhaustive DIVA-search resulted in 72 optimal reconstructions requiring 15 dispersal events. After excluding a single vicariance event between non-Gondwanan landmasses (South America – Eurasia split at node 4), we recovered 60 optimal reconstructions (Figure S3). The early vicariances (Figure S3, nodes 1, 2, 4, 5, 8, 9) can all be explained by break-up events between Gondwanan landmasses that are adjacent (Table S10, "'vicariance (1)", nodes 2, 5, 8, 9), or separated only by a land mass where frogs are currently absent (i.e., implying extinction, but for an obvious reason), such as Antarctica or the Kerguelen Plateau (Table S10, nodes 1 and 4).

Under a strict interpretation, the Madagascar-Eurasia clades (Figure S3, nodes 3, 6, 7) require three contemporary transoceanic dispersals directly from Madagascar to Eurasia (circumventing the intervening Indian Subcontinent). Since this is extremely unlikely in frogs, we suggest that the contemporary, repeated pattern of area relationships between Madagascar and Eurasia is a case of 'mass coherent dispersal' (see manuscript). Since this also involves extinction on India, we performed an additional DIVA analysis to examine the early dispersals and vicariances under this interpretation (Figure S4). This was done by forcing the three Madagascar-Eurasia splits to represent Madagascar-India vicariances, *i.e.*, by coding Eurasian terminals as Indian ones. This constrained analysis resulted in vicariance events at the same nodes, and with very similar ancestral distribution patterns (Figure S4, Table S10, "vicariance (2)", nodes 1, 2, 4, 5, 8, 9). This analysis does not reconstruct any vicariance between non-Gondwanan landmasses, and leads to the same geological interpretation of the six unconstrained vicariance events (Table S10).

**4. References**
