# Supplementary figures and images for: Late Cretaceous Vicariance in Gondwanan Amphibians
Source: PLoS One. 2006 Dec 20;1(1):e74. doi: 10.1371/journal.pone.0000074 (PMC1762348; doi:10.1371/journal.pone.0000074)

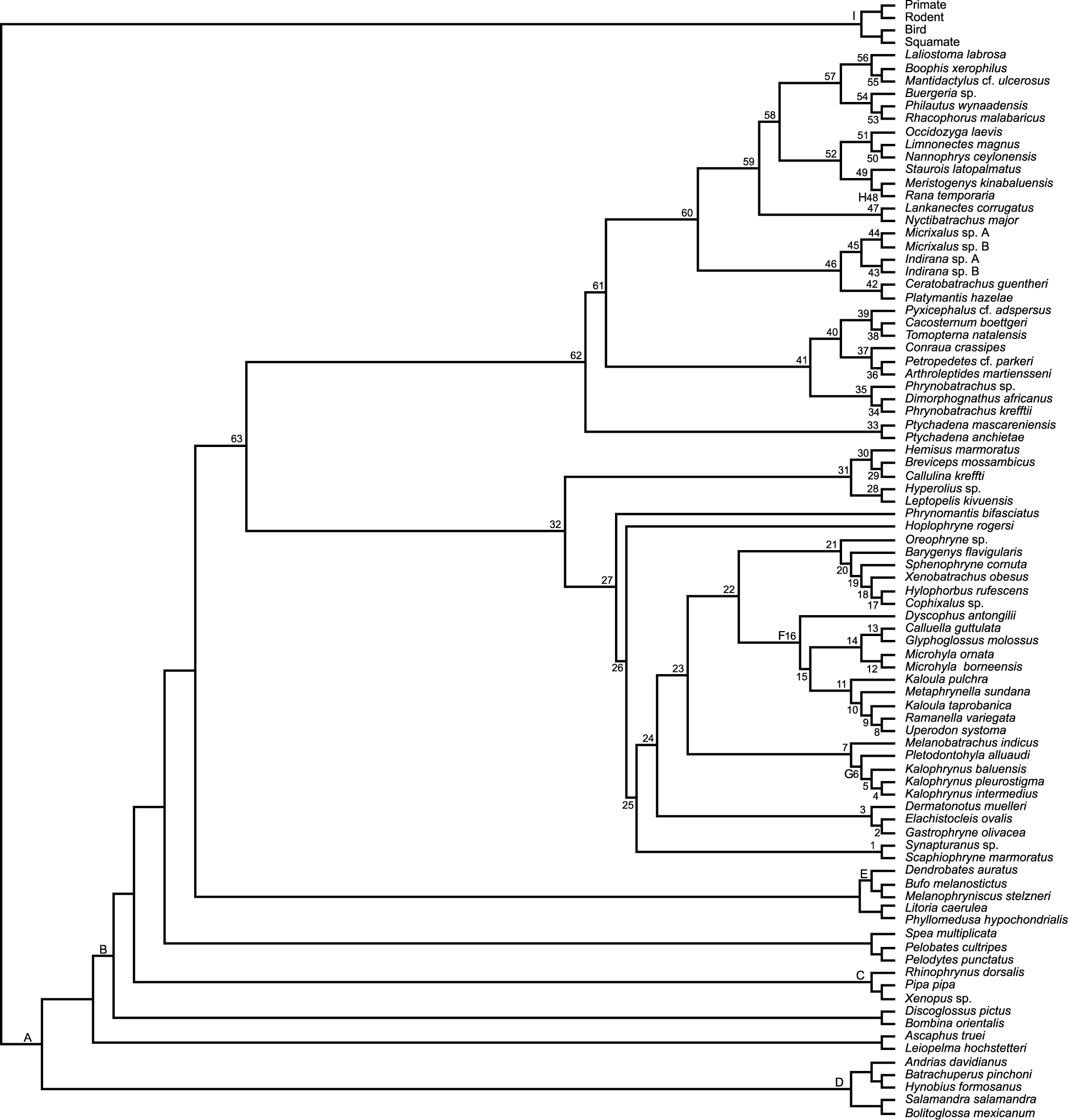

Supplement: Figure S1 — Phylogenetic tree used for the divergence time analyses. The natatanuran and microhylid clades are resolved according to the ML topologies obtained from their respective data sets (Figure 1). Outgroup divergences are resolved according to previous phylogenetic evidence (see Methods). Numbers at ingroup nodes are cross-referenced in Table S9, Letters indicate calibration points. (11.84 MB TIF) [file pone.0000074.s002.tif]

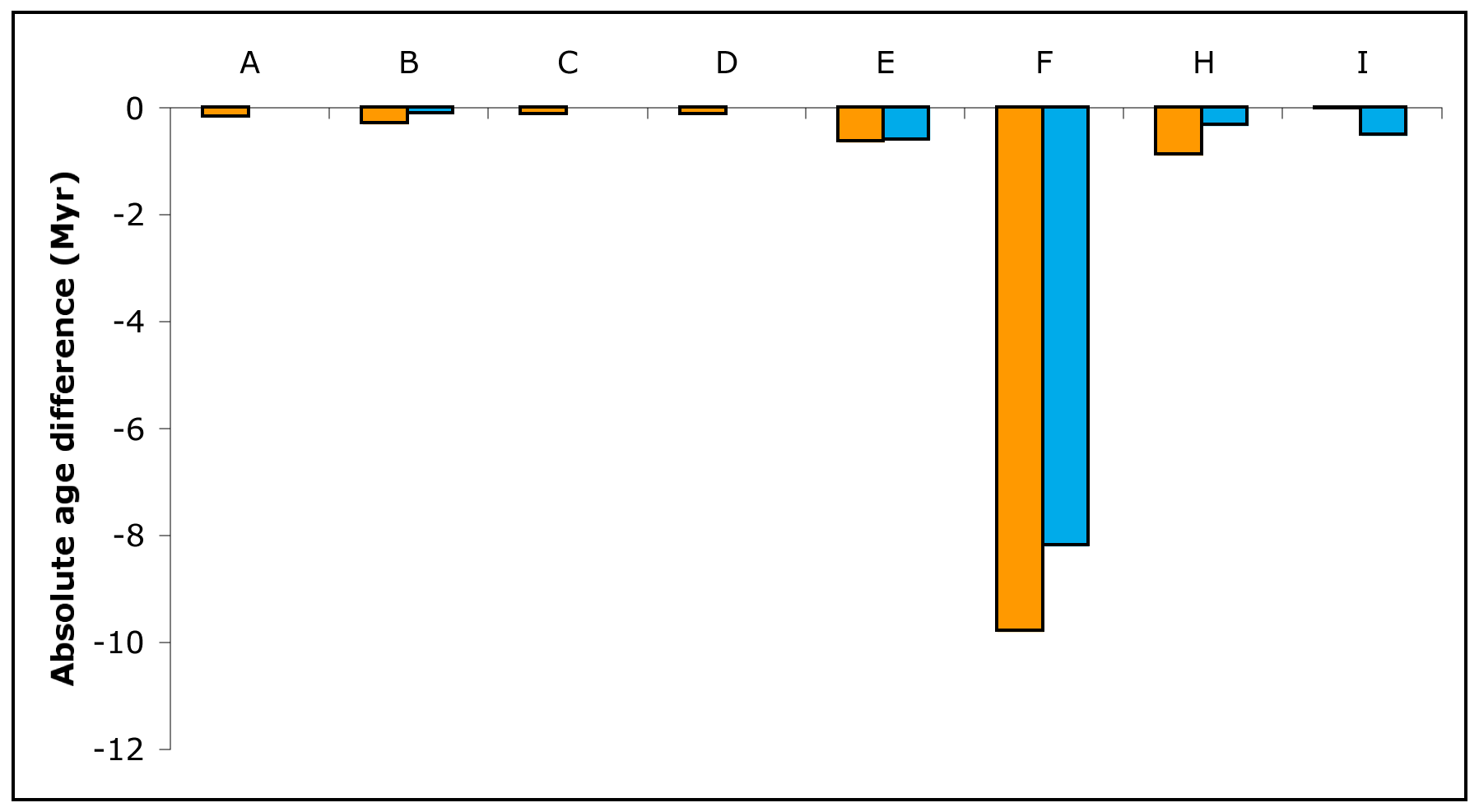

Supplement: Figure S2 — Effect of excluding individual calibration points on divergence time estimates. Letters refer to excluded calibration points and correspond to Tables S8 and S9. The bars represent mean age differences, obtained with the TK-method (orange) and the PL method (blue), compared to the analysis including all calibration points except G. (5.34 MB TIF) [file pone.0000074.s003.tif]

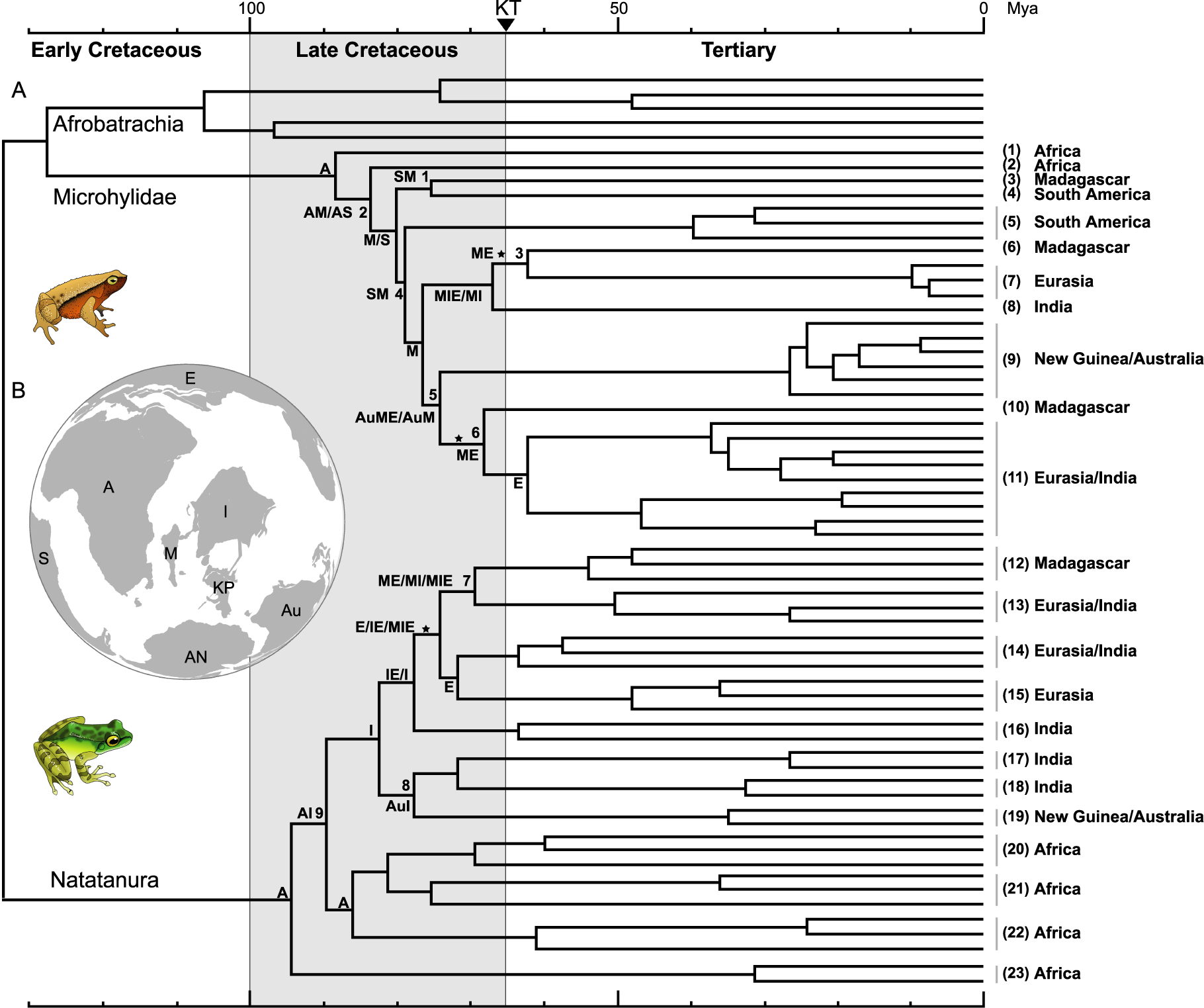

Supplement: Figure S3 — DIVA-reconstruction of ancestral distribution areas. Letter codes at internal nodes correspond to landmasses depicted on the inset globe. (8.35 MB TIF) [file pone.0000074.s004.tif]

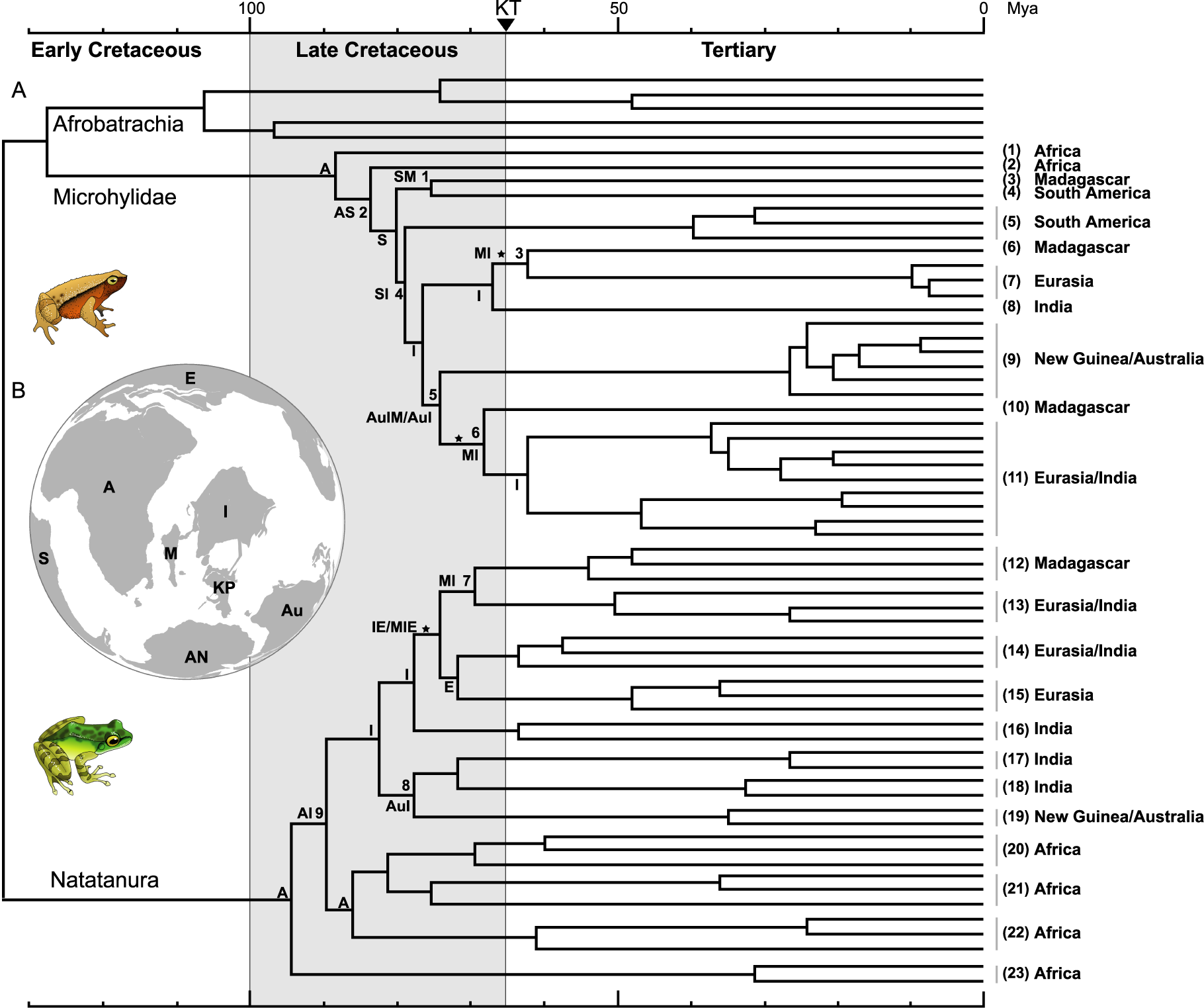

Supplement: Figure S4 — DIVA-reconstruction of ancestral distribution areas, assuming three divergences on Indo-Madagascar. Reconstruction of ancestral distributions under the assumption that nodes 3, 6 and 7 represent vicariance events related to India-Madagascar break-up. Letter codes at internal nodes correspond to landmasses depicted on the inset globe. (8.35 MB TIF) [file pone.0000074.s005.tif]
