# Supplementary material for: Late Cretaceous Vicariance in Gondwanan Amphibians
Source: PLoS One. 2006 Dec 20;1(1):e74. doi: 10.1371/journal.pone.0000074 (PMC1762348; doi:10.1371/journal.pone.0000074)
Supplement: Table S1 — Summary of the sequence data for all sampled nuclear and mitochondrial gene fragments and the total dataset in Microhylidae. (0.31 MB DOC) [file pone.0000074.s006.doc]

| Gene fragment | CXCR-4 | ***Ncx-1*** | Rag-1 | *16S* | Total |
| --- | --- | --- | --- | --- | --- |
| N° of aligned positions | 684 | 1279 | 555 | 576 | 3094 |
| N° of analyzed positions | 651 | 1261 | 536 | 417 | 2865 |
| N° of varying positions | 311 | 472 | 249 | 162 | 1194 |
| N° of parsimony informative positions | 229 | 379 | 203 | 117 | 928 |
