# Supplementary material for: Late Cretaceous Vicariance in Gondwanan Amphibians
Source: PLoS One. 2006 Dec 20;1(1):e74. doi: 10.1371/journal.pone.0000074 (PMC1762348; doi:10.1371/journal.pone.0000074)
Supplement: Table S2 — Taxa included in the Microhylidae dataset, with voucher numbers and origin. (0.34 MB DOC) [file pone.0000074.s007.doc]

| Taxonomy | |  |  |  |
| --- | --- | --- | --- | --- |
| Frost et al., 2006 | Dubois, 2005 | Species | Voucher | Origin |
| **INGROUP** | | | | |
| Microhylidae: Asterophryinae | Microhylidae: Asterophryinae | Barygenys flavigularis | CEBB 47916 | Papua New Guinea |
| Microhylidae: Asterophryinae | Microhylidae: Asterophryinae | *Hylophorbus rufescens* | CEBB 45810 | Papua New Guinea |
| Microhylidae: Asterophryinae | Microhylidae: Asterophryinae | *Xenobatrachus obesus* | CEBB 47591 | Papua New Guinea |
| Microhylidae: Microhylinae | Microhylidae: Calluellinae | *Calluella guttulata* | FMNH 252956 | Vietnam |
| Microhylidae: Cophylinae | Microhylidae: Cophylinae | *Plethodontohyla alluaudi* | AY 571661 | Madagascar |
| Microhylidae: Dyscophinae | Microhylidae: Dyscophinae | *Dyscophus antongilii* | VUB 0888 | Madagascar |
| Microhylidae: Asterophryinae | Microhylidae: Genyophryninae | *Cophixalus* sp. | TNHC 0813CCA | Papua New Guinea |
| Microhylidae: Asterophryinae | Microhylidae: Genyophryninae | *Oreophryne* sp*.* | CEBB 45779 | Papua New Guinea |
| Microhylidae: Asterophryinae | Microhylidae: Genyophryninae | *Sphenophryne cornuta* | CEBB 46144 | Papua New Guinea |
| Microhylidae: Melanobatrachinae | Microhylidae: Hoplophryninae | *Hoplophryne rogersi* | MTSN 5158 | Tanzania |
| Microhylidae: Gastrophryninae | Microhylidae: Microhylinae | *Dermatonotus muelleri* | VUB 0988 | South America |
| Microhylidae: Gastrophryninae | Microhylidae: Microhylinae | *Elachistocleis ovalis* | TNHC DCC3301 | Brasil |
| Microhylidae: Gastrophryninae | Microhylidae: Microhylinae | *Gastrophryne olivacea* | TNHC DCC3106 | U.S.A. |
| Microhylidae | Microhylidae: Microhylinae | *Glyphoglossus molossus* | FMNH 257351 | Cambodia |
| Microhylidae | Microhylidae: Microhylinae | *Kalophrynus baluensis* | VUB0617 | Malaysia |
| Microhylidae | Microhylidae: Microhylinae | *Kalophrynus intermedius* | VUB0604 | Malaysia |
| Microhylidae | Microhylidae: Microhylinae | *Kalophrynus pleurostigma* | VUB 0601 | Malaysia |
| Microhylidae: Microhylinae | Microhylidae: Microhylinae | *Kaloula taprobanica* | VUB0102 | Sri Lanka |
| Microhylidae: Microhylinae | Microhylidae: Microhylinae | *Kaloula pulchra* | VUB0677 | Malaysia |
| Microhylidae: Melanobatrachinae | Microhylidae: Microhylinae | *Melanobatrachus indicus* | VUB 1354 | India |
| Microhylidae | Microhylidae: Microhylinae | *Metaphrynella sundana* | VUB 0646 | Malaysia |
| Microhylidae: Microhylinae | Microhylidae: Microhylinae | *Microhyla borneensis* | VUB0588 | Malaysia |
| Microhylidae: Microhylinae | Microhylidae: Microhylinae | *Microhyla ornata* | VUB 0066 | India |
| Microhylidae | Microhylidae: Microhylinae | *Ramanella variegata* | VUB1342 | India |
| Microhylidae | Microhylidae: Microhylinae | *Synapturanus* sp*.* | NHM MW1004 | Guyana |
| Microhylidae: Microhylinae | Microhylidae: Microhylinae | Uperodon systoma | VUB1151 | Indian subcontinent |
| Microhylidae | Microhylidae: Phrynomerinae | *Phrynomantis bifasciatus* | VUB 0541 | Subsaharan Africa |
| Microhylidae: Scaphiophryninae | Microhylidae: Scaphiophryninae | *Scaphiophryne marmoratus* | VUB 0540 | Madagascar |
| **OUTGROUP** | | | | |
| Brevicipitidae | Brevicipitidae: Brevicipitinae | Breviceps mossambicus | VUB 1031 | Subsaharan Africa |
| Brevicipitidae | Brevicipitidae: Brevicipitinae | *Callulina kreffti* | TNHC 62491 | Tanzania |
| Hemisotidae | Brevicipitidae: Hemisotinae | *Hemisus marmoratus* | CAS 214843 | Kenya |
| Hyperoliidae | Brevicipitidae: Hyperoliinae | *Hyperolius* sp. | VUB 0924 | Kenya |
| Arthroleptidae: Leptopelinae | Brevicipitidae: Leptopelinae | *Leptopelis kivuensis* | CAS 201700 | Uganda |
| Mantellidae: Mantellinae | Ranidae: Mantellinae | *Mantidactylus* cf*. ulcerosus* | ZFMK 66659 | Madagascar |
| Ptychadenidae | Ranidae: Ptychadeninae | *Ptychadena anchietae* | VUB 0958 | Kenya |
| Ranidae | Ranidae: Raninae | *Rana temporaria* | VUB 0920 | Belgium |
| Rhacophoridae: Rhacophorinae | Ranidae: Rhacophorinae | *Philautus wynaadensis* | VUB 0070 | India |
