# Supplementary material for: Late Cretaceous Vicariance in Gondwanan Amphibians
Source: PLoS One. 2006 Dec 20;1(1):e74. doi: 10.1371/journal.pone.0000074 (PMC1762348; doi:10.1371/journal.pone.0000074)
Supplement: Table S3 — Summary of the sequence data for all sampled nuclear and mitochondrial gene fragments and the total dataset in Natatanura. (0.31 MB DOC) [file pone.0000074.s008.doc]

| Gene fragment | CXCR-4 | ***Ncx-1*** | Rag-1 | *Rhod-1* | Rhod-4 | *Tyr* | *16S* | *12V16* | Total |
| --- | --- | --- | --- | --- | --- | --- | --- | --- | --- |
| N° of aligned positions | 673 | 1285 | 555 | 315 | 175 | 534 | 599 | 1501 | 5637 |
| N° of analyzed positions | 586 | 1201 | 537 | 315 | 175 | 516 | 369 | 747 | 4446 |
| N° of varying positions | 272 | 455 | 251 | 135 | 70 | 282 | 160 | 408 | 2033 |
| N° of parsimony informative positions | 218 | 374 | 209 | 98 | 46 | 235 | 130 | 308 | 1618 |
