# Supplementary material for: Late Cretaceous Vicariance in Gondwanan Amphibians
Source: PLoS One. 2006 Dec 20;1(1):e74. doi: 10.1371/journal.pone.0000074 (PMC1762348; doi:10.1371/journal.pone.0000074)
Supplement: Table S4 — Taxa included in the Natatanura dataset, with voucher numbers and origin. (0.34 MB DOC) [file pone.0000074.s009.doc]

| Taxonomy | |  |  |  |
| --- | --- | --- | --- | --- |
| Frost et al., 2006 | Dubois, 2005 | Species | Voucher | Origin |
| **INGROUP** | | | | |
| Ceratobatrachidae | Ranidae: Ceratobatrachinae | Platymantis hazelae | CMNH-RSK 3918 | Philippines |
| Ceratobatrachidae | Ranidae: Ceratobatrachinae | *Ceratobatrachus guentheri* | VUB 1017 (SR 5543) | Solomon Islands |
| Petropedetidae | Ranidae: Conrauinae | *Conraua crassipes* | ZFMK 75446 | Tropical Africa |
| Dicroglossidae: Occidozyginae | Ranidae: Dicroglossinae | *Occidozyga laevis* | VUB 0967 (DLSUD 002) | Philippines |
| Dicroglossidae: Dicroglossinae | Ranidae: Dicroglossinae | *Nannophrys ceylonensis* | VUB 0172 | Sri Lanka |
| Dicroglossidae: Dicroglossinae | Ranidae: Dicroglossinae | *Limnonectes magnus* | VUB 0965 | Philippines |
| Nyctibatrachidae | Ranidae: Lankanectinae | *Lankanectes corrugatus* | VUB 0106 | Sri Lanka |
| Mantellidae: Mantellinae | Ranidae: Mantellinae | *Laliostoma labrosa* | ZFMK 66698 | Madagascar |
| Mantellidae: Mantellinae | Ranidae: Mantellinae | *Mantidactylus* cf*. ulcerosus* | ZFMK 66659 | Madagascar |
| Mantellidae: Boophinae | Ranidae: Mantellinae | Boophis xerophilus | ZFMK 66705 | Madagascar |
| Micrixalidae | Ranidae: Micrixalinae | *Micrixalus* sp. A | VUB 0013 | India |
| Micrixalidae | Ranidae: Micrixalinae | *Micrixalus* sp. B | VUB 0268 | India |
| Nyctibatrachidae | Ranidae: Nyctibatrachinae | Nyctibatrachus major | VUB 0006 | India |
| Petropedetidae | Ranidae: Petropedetinae | Arthroleptides martiensseni | CR 10898 | Africa |
| Petropedetidae | Ranidae: Petropedetinae | *Petropedetes* cf*. parkeri* | VUB 0955 (MV) | Subsaharan Africa |
| Phrynobatrachidae | Ranidae: Phrynobatrachinae | Phrynobatrachus krefftii | VUB 1068 (ES 700) | Tanzania |
| Phrynobatrachidae | Ranidae: Phrynobatrachinae | *Phrynobatrachus* sp. | VUB 0953 | South Africa |
| Phrynobatrachidae | Ranidae: Phrynobatrachinae | *Dimorphognathus africanus* | CAS 207779 | Tropical Africa |
| Ptychadenidae | Ranidae: Ptychadeninae | *Ptychadena mascareniensis* | VUB 0957 | Kenya |
| Ptychadenidae | Ranidae: Ptychadeninae | *Ptychadena anchietae* | VUB 0958 | Kenya |
| Pyxicephalidae: Pyxicephalinae | Ranidae: Pyxicephalinae | *Pyxicephalus* cf. *adspersus* | ZFMK 66446 | Africa |
| Pyxicephalidae: Cacosterninae | Ranidae: Pyxicephalinae | *Cacosternum boettgeri* | ZFMK 66705 | Namibia |
| Pyxicephalidae: Cacosterninae | Ranidae: Pyxicephalinae | *Tomopterna natalensis* | ZFMK 68815 | South Africa |
| Ranidae | Ranidae: Raninae | *Staurois latopalmatus* | VUB 0652 | Malaysia |
| Ranidae | Ranidae: Raninae | *Meristogenys kinabaluensis* | VUB 0627 | Malaysia |
| Ranidae | Ranidae: Raninae | *Rana temporaria* | VUB 0920 | Belgium |
| Petropedetidae | Ranidae: Ranixalinae | *Indirana* sp. A | VUB 0037 | India |
| Petropedetidae | Ranidae: Ranixalinae | *Indirana* sp. B | VUB 0223 | India |
| Rhacophoridae: Buergeriinae | Ranidae: Rhacophorinae | *Buergeria* sp. | VUB 0797 | Taiwan |
| Rhacophoridae: Rhacophorinae | Ranidae: Rhacophorinae | Rhacophorus malabaricus | VUB 0001 | India |
| Rhacophoridae: Rhacophorinae | Ranidae: Rhacophorinae | *Philautus wynaadensis* | VUB 0070 | India |
| **OUTGROUP** | | | | |
| Brevicipitidae | Brevicipitidae: Brevicipitinae | *Callulina kreffti* | TNHC 62491 | Tanzania |
| Hemisotidae | Brevicipitidae: Hemisotinae | *Hemisus marmoratus* | CAS 214843 | Kenya |
| Hyperoliidae | Brevicipitidae: Hyperoliinae | *Hyperolius* sp. | VUB 0924 | Kenya |
| Arthroleptidae: Leptopelinae | Brevicipitidae: Leptopelinae | *Leptopelis kivuensis* | CAS 201700 | Uganda |
| Dendrobatidae | Dendrobatidae | *Dendrobates auratus* | VUB 0986 | South America |
| Microhylidae: Asterophryinae | Microhylidae: Genyophryninae | *Cophixalus* sp. | TNHC 51333 | Papua New Guinea |
| Microhylidae: Gastrophryninae | Microhylidae: Microhylinae | *Elachistocleis ovalis* | TNHC DCC3301 | Brasil |
| Microhylidae: Gastrophryninae | Microhylidae: Microhylinae | *Gastrophryne olivacea* | TNHC 61952 | U.S.A. |
| Microhylidae: Microhylinae | Microhylidae: Microhylinae | Kaloula taprobanica | VUB 0102 | Sri Lanka |
| Microhylidae: Microhylinae | Microhylidae: Microhylinae | *Microhyla ornata* | VUB 0066 | India |
