# Supplementary material for: Late Cretaceous Vicariance in Gondwanan Amphibians
Source: PLoS One. 2006 Dec 20;1(1):e74. doi: 10.1371/journal.pone.0000074 (PMC1762348; doi:10.1371/journal.pone.0000074)
Supplement: Table S5 — Taxa with GenBank accession numbers of homologous gene fragments for microhylid species. (0.34 MB DOC) [file pone.0000074.s010.doc]

| **Species** | | **GenBank Accession Number** | | | |
| --- | --- | --- | --- | --- | --- |
|  |  | *Cxcr-4* | *Ncx-1* | *Rag-1* | *16S* |
|  |  |  |  |  |  |
| **MICROHYLIDAE** |  |  |  |  |  |
| *Barygenys* | *flavigularis* | AY948800 | AY948845 | AY948943 | AY948767 |
| *Calluella* | *guttulata* | EF017975 | EF018031 | EF018045 | EF017956 |
| *Cophixalus* | sp. | EF017967 | EF018004 | DQ347276 | DQ347334 |
| *Dermatonotus* | *muelleri* | AY948785 | AY948825 | AY948928 | AY948747 |
| *Dyscophus* | *antongilii* | AY948790 | AY948832 | AY948933 | AY948754 |
| *Elachistocleis* | *ovalis* | EF017969 | EF018006 | DQ347282 | DQ347340 |
| *Gastrophryne* | *olivacea* | EF017968 | EF018005 | DQ347280 | DQ347338 |
| *Glyphoglossus* | *molossus* | AY948777 | AY948812 | AY948920 | AY948734 |
| *Hoplophryne* | *rogersi* | EF017980 | EF018036 | EF018050 | EF017961 |
| *Hylophorbus* | *rufescens* | EF017977 | EF018033 | EF018047 | EF017958 |
| *Kalophrynus* | *baluensis* | EF017972 | EF018028 | EF018042 | EF017953 |
| *Kalophrynus* | *intermedius* | EF017971 | EF018027 | EF018041 | EF017952 |
| *Kalophrynus* | *pleurostigma* | AY948776 | AY948811 | AY948919 | AY948733 |
| *Kaloula* | *pulchra* | EF017974 | EF018030 | EF018044 | EF017955 |
| *Kaloula* | *taprobanica* | AY948772 | AY948807 | AY948915 | AF249057 |
| *Melanobatrachus* | *indicus* | EF017983 | EF018039 | EF018053 | EF017964 |
| *Metaphrynella* | *sundana* | EF017973 | EF018029 | EF018043 | EF017954 |
| *Microhyla* | *borneensis* | EF017970 | EF018026 | EF018040 | EF017951 |
| *Microhyla* | *ornata* | AY364168 | AY948806 | AY364198 | AF249060 |
| *Oreophryne* | sp. | EF017976 | EF018032 | EF018046 | EF017957 |
| *Phrynomantis* | *bifasciatus* | AY948775 | AY948810 | AY948918 | AY948732 |
| *Plethodontohyla* | *alluaudi* | n.a. | n.a. | AY571661 | AY594112 |
| *Ramanella* | *variegata* | EF017982 | EF018038 | EF018052 | EF017963 |
| *Scaphiophryne* | *marmoratus* | AY364175 | AY523712 | AY364205 | AY364367 |
| *Sphenophryne* | *cornuta* | AY948799 | AY948844 | AY948942 | AY948766 |
| *Synapturanus* | sp. | EF017981 | EF018037 | EF018051 | EF017962 |
| *Uperodon* | *systoma* | EF017979 | EF018035 | EF018049 | EF017960 |
| *Xenobatrachus* | *obesus* | EF017978 | EF018034 | EF018048 | EF017959 |
|  |  |  |  |  |  |
