# Supplementary material for: Late Cretaceous Vicariance in Gondwanan Amphibians
Source: PLoS One. 2006 Dec 20;1(1):e74. doi: 10.1371/journal.pone.0000074 (PMC1762348; doi:10.1371/journal.pone.0000074)
Supplement: Table S6 — Taxa with GenBank accession numbers of homologous gene fragments for natatanuran species. (0.36 MB DOC) [file pone.0000074.s011.doc]

| **Species** | | **GenBank Accession Number** | | | | | | |  |
| --- | --- | --- | --- | --- | --- | --- | --- | --- | --- |
|  |  | *Cxcr-4* | *Ncx-1* | *Rag-1* | *Rhod-1* | *Rhod-4* | *Tyr* | *16S* | *12V16* |
|  |  |  |  |  |  |  |  |  |  |
| **NATATANURA** |  |  |  |  |  |  |  |  |  |
| *Arthroleptides* | *martiensseni* | n.a. | n.a. | DQ347289 | DQ347410 | n.a. | DQ347197 | DQ347346 | DQ347064 |
| *Boophis* | *xerophilus* | AY364179 | EF018013 | AY364209 | AF249104 | AF249136 | AF249167 | AF249038 | DQ346999 |
| *Buergeria* | sp. | AY948778 | AY948813 | DQ347226 | DQ347350 | DQ347067 | DQ347134 | DQ347292 | DQ346989 |
| *Cacosternum* | *boettgeri* | AY948781 | AY948818 | AY948924 | DQ347358 | DQ347074 | DQ347141 | DQ347299 | DQ347007 |
| *Ceratobatrachus* | *guentheri* | AY948788 | AY948830 | DQ347272 | DQ347391 | DQ347111 | DQ347179 | DQ347330 | DQ347046 |
| *Conraua* | *crassipes* | EF017991 | EF018016 | DQ347244 | DQ347364 | DQ347081 | DQ347148 | DQ347305 | DQ347015 |
| *Dimorphognathus* | *africanus* | EF017996 | EF018021 | DQ347259 | DQ347378 | DQ347097 | DQ347164 | DQ347319 | DQ347031 |
| *Indirana* | sp. A | EF017984 | EF018007 | DQ347204 | AF249122 | AF249154 | AF249185 | AF249051 | DQ346961 |
| *Indirana* | sp. B | EF017985 | EF018009 | DQ347215 | AF249123 | AF249155 | AF249186 | AF249064 | DQ346977 |
| *Laliostoma* | *labrosa* | AY948780 | AY948816 | AY948923 | AF249106 | AF249138 | AF249169 | AF249037 | DQ346998 |
| *Lankanectes* | *corrugatus* | AY948773 | AY948808 | AY948916 | AF249115 | AF249147 | AF249178 | AF249043 | DQ346971 |
| *Limnonectes* | *magnus* | EF017994 | EF018019 | DQ347252 | DQ347373 | DQ347090 | DQ347157 | DQ347314 | DQ347022 |
| *Mantidactylus* | cf. *ulcerosus* | AY948779 | AY948815 | AY948922 | AF249102 | AF249134 | AF249165 | AF249035 | DQ346996 |
| *Meristogenys* | *kinabaluensis* | AY364176 | AY523704 | AY364206 | AY322292 | AY322317 | AY322357 | AY322267 | DQ346983 |
| *Micrixalus* | sp. A | AY948771 | AY948804 | AY948914 | AF249120 | AF249152 | AF249183 | AF249056 | DQ346959 |
| *Micrixalus* | sp. B | EF017986 | EF018010 | DQ347216 | AF249021 | AF249153 | AF249184 | AF249041 | DQ346978 |
| *Nannophrys* | *ceylonensis* | AY948774 | AY948809 | AY948917 | AF249112 | AF249144 | AF249175 | AF249047 | DQ346975 |
| *Nyctibatrachus* | cf. *major* | AY948770 | AY948803 | AY948913 | AF249113 | AF249145 | AF249176 | AF249052 | DQ346958 |
| *Occidozyga* | *laevis* | EF017995 | EF018020 | DQ347254 | AY322300 | AY322329 | AY322342 | AY322262 | DQ347024 |
| *Petropedetes* | cf*. parkeri* | AY364183 | AY948819 | AY364213 | AY364394 | DQ347080 | DQ347147 | AY364369 | DQ347014 |
| *Philautus* | *wynaadensis* | AY364169 | EF018008 | AY364199 | AF249127 | AF249159 | AF249190 | AF249059 | DQ346966 |
| *Phrynobatrachus* | *krefftii* | EF017997 | EF018022 | DQ347284 | DQ347403 | DQ347124 | DQ347192 | DQ347342 | DQ347059 |
| *Phrynobatrachus* | sp. | EF017990 | EF018015 | DQ347242 | DQ347362 | DQ347078 | DQ347145 | DQ347303 | DQ347012 |
| *Platymantis* | *hazelae* | EF017993 | EF018018 | DQ347248 | DQ347369 | DQ347086 | DQ347153 | DQ347310 | DQ347019 |
| *Ptychadena* | *anchietae* | AY948782 | AY948820 | AY948925 | DQ347366 | DQ347083 | DQ347150 | DQ347307 | DQ347017 |
| *Ptychadena* | *mascareniensis* | EF017992 | EF018017 | DQ347245 | DQ347365 | DQ347082 | DQ347149 | DQ347306 | DQ347016 |
| *Pyxicephalus* | cf. *adspersus* | n.a. | n.a. | DQ347243 | DQ347363 | DQ347079 | DQ347146 | DQ347304 | DQ347013 |
| *Rana* | *temporaria* | EF017988 | EF018012 | DQ347231 | AF249119 | AF249151 | AF249182 | AF249048 | DQ346993 |
| *Rhacophorus* | *malabaricus* | AY948769 | AY948802 | DQ347202 | AF249125 | AF249157 | AF249188 | AF249050 | DQ346957 |
| *Staurois* | *latopalmatus* | EF017987 | EF018011 | DQ347221 | AY322290 | AY322327 | AY322359 | AY322257 | DQ346984 |
| *Tomopterna* | *natalensis* | EF017989 | EF018014 | DQ347239 | DQ347359 | DQ347075 | DQ347142 | DQ347300 | DQ347008 |
|  |  |  |  |  |  |  |  |  |  |
