# Supplementary material for: Late Cretaceous Vicariance in Gondwanan Amphibians
Source: PLoS One. 2006 Dec 20;1(1):e74. doi: 10.1371/journal.pone.0000074 (PMC1762348; doi:10.1371/journal.pone.0000074)
Supplement: Table S7 — Taxa with GenBank accession numbers of homologous gene fragments for outgroup species. (0.37 MB DOC) [file pone.0000074.s012.doc]

| **Species** | | **GenBank Accession Number** | | | | | | |  |
| --- | --- | --- | --- | --- | --- | --- | --- | --- | --- |
|  |  | *Cxcr-4* | *Ncx-1* | *Rag-1* | *Rhod-1* | *Rhod-4* | *Tyr* | *16S* | *12V16* |
|  |  |  |  |  |  |  |  |  |  |
| **OUTGROUP** |  |  |  |  |  |  |  |  |  |
| *Breviceps* | *mossambicus* | EF017965 | EF018002 | EF018056 | n.a. | n.a. | n.a. | EF017947 | n.a. |
| *Callulina* | *kreffti* | EF017966 | EF018003 | DQ347281 | DQ347400 | DQ347121 | DQ347189 | DQ347339 | DQ347056 |
| *Cophixalus* | sp. | EF017967 | EF018004 | DQ347276 | DQ347395 | DQ347115 | DQ347183 | DQ347334 | DQ347050 |
| *Dendrobates* | *auratus* | AY364184 | AY948823 | AY364214 | AY364395 | DQ347093 | DQ347160 | AY364370 | DQ347026 |
| *Elachistocleis* | *ovalis* | EF017969 | EF018006 | DQ347282 | DQ347401 | DQ347122 | DQ347190 | DQ347340 | DQ347057 |
| *Gastrophryne* | *olivacea* | EF017968 | EF018005 | DQ347280 | DQ347399 | DQ347120 | DQ347188 | DQ347338 | DQ347055 |
| *Hemisus* | *marmoratus* | AY364186 | AY948827 | AY364216 | DQ347380 & AY364397 | DQ347098 | DQ347166 | AY364372 | DQ347033 |
| *Hyperolius* | sp. | AY364178 | AY948814 | AY364208 | AF249098 & AY364392 | AF249130 | AF249161 | AF249033 | DQ346994 |
| *Kaloula* | *taprobanica* | AY948772 | AY948807 | AY948915 | AF249100 | AF249132 | AF249163 | AF249057 | DQ346970 |
| *Leptopelis* | *kivuensis* | AY364181 | AY523717 | AY364211 | AY322275 | AY322328 | AY322335 | AY322245 | DQ347005 |
| *Mantidactylus* | cf. *ulcerosus* | AY948779 | AY948815 | AY948922 | AF249102 | AF249134 | AF249165 | AF249035 | DQ346996 |
| *Microhyla* | *ornata* | AY364168 | AY948806 | AY364198 | AF249099 & AY364383 | AF249131 | AF249162 | AF249060 | DQ346965 |
| *Philautus* | *wynaadensis* | AY364169 | EF018008 | AY364199 | AF249127 | AF249159 | AF249190 | AF249059 | DQ346966 |
| *Ptychadena* | *anchietae* | AY948782 | AY948820 | AY948925 | DQ347366 | DQ347083 | DQ347150 | DQ347307 | DQ347017 |
| *Rana* | *temporaria* | EF017988 | EF018012 | DQ347231 | AF249119 | AF249151 | AF249182 | AF249048 | DQ346993 |
|  |  |  |  |  |  |  |  |  |  |
| *Andrias* | *davidianus* | AY948801 | AY948847 | AY948944 | n.a. | n.a. | n.a. | AJ492192 | n.a. |
| *Ascaphus* | *truei* | AY523695 | AY523731 | AY523751 | n.a. | n.a. | n.a. | AY523780 | n.a. |
| *Batrachuperus* | *pinchoni* | EF017998 | EF018023 | EF018054 | n.a. | n.a. | n.a. | EF017948 | n.a. |
| *Bolitoglossa* | *mexicanum* | EF018000 | EF018025 | EF018057 | n.a. | n.a. | n.a. | EF017950 | n.a. |
| *Bombina* | *orientalis* | AY364177 | AY523715 | AY364207 | n.a. | n.a. | n.a. | AY364368 | n.a. |
| *Bufo* | *melanostictus* | AY364167 | AY948805 | AY364197 | n.a. | n.a. | n.a. | AF249061 | n.a. |
| *Discoglossus* | *pictus* | AY364172 | AY523708 | AY364202 | n.a. | n.a. | n.a. | AY364364 | n.a. |
| *Hynobius* | *formosanus* | EF01801 | AY523732 | DQ347285 | n.a. | n.a. | n.a. | AY523782 | n.a. |
| *Leiopelma* | *hochstetteri* | AY523696 | AY523734 | AY523753 | n.a. | n.a. | n.a. | AY523784 | n.a. |
| *Litoria* | *caerulea* | AY948783 | AY948821 | AY948926 | n.a. | n.a. | n.a. | AY948743 | n.a. |
| *Melanophryniscus* | *stelzneri* | AY948784 | AY948822 | AY948927 | n.a. | n.a. | n.a. | AY948744 | n.a. |
| *Pelobates* | *cultripes* | AY364171 | AY523707 | AY364201 | n.a. | n.a. | n.a. | AY364363 | n.a. |
| *Pelodytes* | *punctatus* | AY364173 | AY523709 | AY364203 | n.a. | n.a. | n.a. | AY364365 | n.a. |
| *Phyllomedusa* | *hypochondrialis* | AY948786 | AY948826 | AY948929 | n.a. | n.a. | n.a. | AY948748 | n.a. |
| *Pipa* | *pipa* | AY364174 | AY523711 | AY364204 | n.a. | n.a. | n.a. | AY364366 | n.a. |
| *Rhinophrynus* | *dorsalis* | AY523699 | AY523722 | AY523747 | n.a. | n.a. | n.a. | AY523781 | n.a. |
| *Salamandra* | *salamandra* | EF017999 | EF018024 | DQ347230 | n.a. | n.a. | n.a. | EF017949 | n.a. |
| *Spea* | *multiplicata* | AY523701 | AY523724 | AY523749 | n.a. | n.a. | n.a. | AY523786 | n.a. |
| *Xenopus* | sp. | AY523691 | AY523716 | AY523743 | n.a. | n.a. | n.a. | AY523771 | n.a. |
|  |  |  |  |  |  |  |  |  |  |
