# Supplementary material for: Late Cretaceous Vicariance in Gondwanan Amphibians
Source: PLoS One. 2006 Dec 20;1(1):e74. doi: 10.1371/journal.pone.0000074 (PMC1762348; doi:10.1371/journal.pone.0000074)
Supplement: Table S8 — Calibration points used in this study. (0.34 MB DOC) [file pone.0000074.s013.doc]

| **Node** | | **Fossil or paleogeographic event** | **Reference** | **Age constraint** (Mya) |
| --- | --- | --- | --- | --- |
| **A** | Split Anura - Caudata | †*Triadobratrachus massinotii* (Induan, Early Triassic) | [58] | > 245.0 |
| **B** | Stem-origin of Costata | †*Eodiscoglossus oxoniensis* (Bathonian, Middle Jurassic) | [59] | > 164.0 |
| **C** | Split Rhinophrynidae - Pipidae | †*Rhadinosteus parvus* (Kimmeridgian, Late Jurassic) | [60] | > 151.0 |
| **D** | Split Cryptobranchidae - Hynobiidae | †*Chunerpeton tianyiensis*.  (Daohugou deposits, Jurassic/Cretaceous boundary) | [35,37,38] | > 145.5 |
| **E** | Stem origin of Bufonidae | Oldest †Bufonidae sp. (Thanetian, Late Paleocene) | [61] | > 54.8 |
| **F** | Split *Dyscophus* - Microhylinae | Youngest postulated connection India–Madagascar | [45,46,47] | > 65.0 |
| **G** | Split *Plethodontohyla* - Kalophryninae | Youngest postulated connection India–Madagascar | [45,46,47] | > 65.0 |
| **H** | Stem-origin of *Rana* | Oldest †*Rana* sp. (Rupelian, E. Oligocene) | [48] | > 28.5 |
| **I** | Split Birds - Mammals | Earliest crown-group diapsids and synapsids | [53] | 332.3 > > 306.1 |
