# Supplementary material for: Late Cretaceous Vicariance in Gondwanan Amphibians
Source: PLoS One. 2006 Dec 20;1(1):e74. doi: 10.1371/journal.pone.0000074 (PMC1762348; doi:10.1371/journal.pone.0000074)
Supplement: Table S10 — Dating estimates (mya) for Microhylidae and Natatanura, with DIVA reconstruction of vicariance events in their early evolution. For both the TK- and PL-method, the ages represent the 95% confidence intervals of the analysis that includes all calibration points except G. Vicariance (1) represents the strict analysis; Vicariance (2) represents the analysis with the three India-Eurasia splits constrained as India-Madagascar. The two DIVA reconstructions represent the actual distribution of the frogs, while the associated continental break-up is the presumed geological unit at the moment of break-up, based on our divergence times. Node numbers and colors associated with break-up correspond with figure 2. Afr = Africa, Ind = India, SAm = South America, Mad = Madagascar, Aus = Australia-New Guinea, Eur = Eurasia, KP = Kerguelen Plateau. (0.33 MB DOC) [file pone.0000074.s015.doc]

| **Node** | **Age (TK)** | **Age (PL)** | **Vicariance (1)** | **Vicariance (2)** | **Associated continental break-up** |
| --- | --- | --- | --- | --- | --- |
|  |  |  |  |  |  |
| 9 | [74.2, 107.7] | [61.7, 75.0] | Afr - Ind | Afr - Ind | Africa <--> Indo-Madagascar (blue) |
| 2 | [74.2, 97.1] | [68.2, 74.9] | Afr - SAm | Afr - SAm | Africa <--> South America (yellow) |
|  |  |  | *or* Afr - Mad |  | Africa <--> Indo-Madagascar (blue) |
| 1 | [65.4, 88.1] | [51.7, 69.1] | SAm - Mad | SAm - Mad | S. America-Antarctica <--> Indo-Madagascar (disappearance KP) (green) |
| 4 | [70.4, 91.4] | [66.8, 71.4] | SAm - Mad | SAm - Ind | S. America-Antarctica <--> Indo-Madagascar (disappearance KP) (green) |
| 8 | [63.1, 95.0] | [52.3, 68.5] | Aus - Ind | Aus - Ind | Australia <--> Indo-Madagascar (orange) |
| 5 | [66.7, 84.5] | [65.2, 68.5] | Aus - Mad | Aus - Ind | Australia <--> Indo-Madagascar (orange) |
|  |  |  | *or* Aus - MadEur | *or* Aus - IndMad |  |
| 3 | [48.7, 75.8] | [49.6, 66.1] | Mad - Eur | Mad - Ind | Madagascar <--> India (Seychelles) (purple) |
| 6 | [65.1, 77.6] | [65.0, 65.0] | Mad - Eur | Mad - Ind | Madagascar <--> India (Seychelles) (purple) |
| 7 | [56.0, 86.2] | [50.5, 62.8] | Mad - Eur | Mad - Ind | Madagascar <--> India (Seychelles) (purple) |
|  |  |  | *or* Mad - Ind |  |  |
|  |  |  | *or* Mad - EurInd |  |  |
|  |  |  |  |  |  |
